# The spectral, spatial and contrast sensitivity of human polarization pattern perception

**DOI:** 10.1038/s41598-017-16873-6

**Published:** 2017-11-29

**Authors:** Gary P. Misson, Stephen J. Anderson

**Affiliations:** 1 Department of Ophthalmology, South Warwickshire NHS Foundation Trust, Warwick Hospital, Lakin Road, Warwick, CV34 5BW UK; 2 School of Life and Health Sciences, Aston University, Birmingham, B4 7ET UK

## Abstract

It is generally believed that humans perceive linear polarized light following its conversion into a luminance signal by diattenuating macular structures. Measures of polarization sensitivity may therefore allow a targeted assessment of macular function. Our aim here was to quantify psychophysical characteristics of human polarization perception using grating and optotype stimuli defined solely by their state of linear polarization. We show: (i) sensitivity to polarization patterns follows the spectral sensitivity of macular pigment; (ii) the change in sensitivity across the central field follows macular pigment density; (iii) polarization patterns are identifiable across a range of contrasts and scales, and can be resolved with an acuity of 15.4 cycles/degree (0.29 logMAR); and (iv) the human eye can discriminate between areas of linear polarization differing in electric field vector orientation by as little as 4.4°. These findings, which support the macular diattenuator model of polarization sensitivity, are unique for vertebrates and approach those of some invertebrates with a well-developed polarization sense. We conclude that this sensory modality extends beyond Haidinger’s brushes to the recognition of quantifiable spatial polarization-modulated patterns. Furthermore, the macular origin and sensitivity of human polarization pattern perception makes it potentially suitable for the detection and quantification of macular dysfunction.

## Introduction

Many animal species are sensitive not only to light intensity and wavelength but also to the polarization information contained within light^1^. Linearly polarized light consists of electromagnetic waves with parallel electric field vectors (**e**-vector), and the ability of some animals to discriminate between different **e**-vector orientations is thought to aid their vision, navigation, camouflage, predation and social communication (reviewed in Horvath^2^). The biophysical basis by which this is achieved varies between species and is not fully understood, although it is generally agreed that polarization vision has its origin in front-end photoreceptors that exhibit a differential polarization response^3,4^. In some species, sensitivity to polarized light may be further enhanced through the operation of higher-order polarization-opponent and spatial integration mechanisms^5^.

Whilst humans lack any dedicated receptors or higher-order neurons for polarization vision, they are capable of perceiving linearly polarized light^6^. It has long been believed that this particular human sense was rudimentary and limited to the perception of Haidinger’s ‘brushes’^7,8^, the short-lived hourglass-shaped pattern of orthogonal yellow and blue hues perceived near the locus of fixation when one’s gaze is directed at a uniform field of linearly polarized white light. The phenomenon is wavelength-sensitive, closely following the absorption spectrum of macular pigment^8–10^, and is sensitive to corneal birefringence^11–13^.

More recent evidence^14^ shows that human polarization sensitivity is not limited to the perception of Haidinger’s brushes: non-uniform polarization-modulated stimuli yield percepts that are markedly different from the classic ‘brush’ configuration seen with uniform fields of polarized light. This ability can best be described as *polarization pattern perception*, a particular characteristic of which is the preservation of edge boundaries between two adjacent areas of different linear polarization. This edge-preserving property allows the design of stimuli that could be utilised in a full psychophysical characterisation of human polarization vision, including one- and two-dimensionally modulated patterns and optotypes.

The precise mechanism of human polarization perception is unclear. As humans do not possess any specialised polarization-sensitive mechanisms^15^, it is hypothesised that this perceptual feat originates from the conversion of linearly polarized light into a luminance contrast signal by a diattenuating structure (or structures) within the eye^6^. Various candidate structures have been proposed, including the arrangement of absorbing macular pigment molecules within radially symmetric photoreceptor cell axons^7,16^, the birefringent properties of the photoreceptor cell axons^17^, and the distribution and cylindrical geometry of short-wavelength sensitive cones^18^. Evidence that human polarization vision is tied in some way to macular pigment includes: (i) the near perfect match between the wavelength of light yielding peak sensitivity to Haidinger’s brushes and the peak absorption characteristics of macular pigment^10,19,20^; and (ii) the confinement of Haidinger’s brushes to the central two to three degrees of the visual field^7,8^, although there is no quantitative data supporting this.

Human polarization perception provides no known biological or behavioural advantage. However, the intimate relationship that appears to exist between macular pigment and polarized light perception may afford the opportunity to develop novel and clinically useful measures of macular function. This is important because macular disease is one of the major causes of blindness throughout the developed world, and treatment is currently only effective in early stages of disease progression.

Our aim in this paper was to employ polarization-modulated grating patterns and optotypes in order to quantify fully the spectral characteristics, spatial characteristics and resolution of human polarization pattern perception.

## Materials and Methods

The following standard polarization parameters are used in this study: polarization orientation (*ψ*), ellipticity (*χ*)^21^ and degree of polarization (DOP). These are defined, together with calibration methodology, in the Supplementary Material. Other study-specific parameters are defined below.

### Participants

A total of nine adults (5 males and 4 females, aged 27–62 years) consented to participate in the study, with five participants for the spectral sensitivity measures and six for both the visual field and contrast sensitivity measures. All had normal visual fields, normal colour vision and normal or corrected-to-normal monocular logMAR acuities of 0.0 or less. Participants provided informed consent and all procedures were approved by the UK NHS Health Research Authority and Ethics Committee (IRAS no. 224715). All experimental procedures were in accordance with the tenets of the Declaration of Helsinki.

### Polarization stimuli

#### Stimulus generation

Stimuli were generated by computer and displayed on a conventional 5” 800 × 480 pixel HDMI LED-backlit thin-film transistor liquid crystal display (LCD, from Waveshare Electronics, Shenzhen, China). To generate an isoluminant, isochromatic polarization stimulus with a variable polarization angle, the front polarizing filter of the LCD was removed and the resultant delaminated LCD (dLCD) cell was illuminated with an appropriate light source^14^. To assess spectral sensitivity the light source was a constant intensity monochromator, while for all other measures the light source was that of the original LCD, but with a blue filter inserted between the light source and the back polarizing filter – particular details of the light source are reported in the relevant sections below. Note that luminance contrast measures were completed with the front polarizer in place.

#### Stimulus calibration

The polarization output of the dLCD was measured for greyscale values (r = g = b = [0, 255]) using standard polarimetric methods (see Supplementary Material). It was thus possible to assign a state of polarization generated by the dLCD to a given greyscale value. Note that the degree of polarization of all greyscale values exceeded 94%. Polarization ellipticity was low at the ends of the greyscale range (*χ* < 5° for greyscales 0–33 and 220–255), reaching a maximum of 14° for intermediate greyscale values (153). There is a clearly defined non-linear relationship between greyscale and polarization angle orientation ranging from 43° to 130° for greyscale value of 0 to 255 respectively (Supplementary Material, Figure S1a), which allows a precise polarization orientation value generated by the dLCD to be assigned to any particular input greyscale value.

Luminance was measured with a Minolta photometer (model CS100-A), and the gamma-corrected display was linear over the range of contrasts used.

#### Contrast calibration, polarization angle, polarization angle contrast

An image comprising adjacent areas of different greyscales creates a luminance difference on the intact display (LCD) and a polarization difference on the delaminated display (dLCD). For a given pattern the luminance difference on the LCD can be expressed using a conventional luminance contrast measure, such as Weber (C_W_) or Michelson (C_M_) contrast (see below). The same pattern on the dLCD is isoluminant but can be quantified as if displayed on a conventional LCD using a polarization equivalent luminance contrast parameter (polarization equivalent contrast, _PEQ_C). This can be converted to a polarization angle difference (Δ*ψ°*) or an angular contrast value (polarization angle contrast, _P_C = Δ*ψ°*/90°), according to the method outlined in the Supplementary Material.

### Procedure

Details specific to each experiment are outlined in the relevant sections below. The general procedure was to measure contrast thresholds for discriminating the orientation of visual patterns (either square-wave modulated gratings or Landolt C optotypes) using a four-alternate forced-choice (4-AFC) paradigm in a modified version of the Freiburg Visual Acuity and Contrast Test (FrACT Version 3.9.8^22^). FrACT uses the best parameter estimation by a sequential testing (best PEST) algorithm for adaptive threshold determination, with each run consisting of 24 trials. Stimulus orientation was randomised between trials, and participants made their response selection at their own pace using a dedicated, hand-held directional keypad. Auditory feedback was given for an incorrect response. For all measures the ambient room illumination was 42 lux.

The stimulus display screen was mounted on an optical bench and viewed at a distance of 50 cm, with the participant’s head stabilized using a chin and forehead rest. This arrangement allowed viewing normal to the screen, eliminating the possibility of luminance confounds from oblique viewing. If required, a participant’s refractive error was corrected to 50 cm using non-birefringent glass lenses. At this distance, the full display screen subtended 12° horizontally by 8° vertically.

The contrast of grating patterns was defined as Michelson contrast (C_M_):

$${{\rm{C}}}_{{\rm{M}}}=({\rm{Lmax}}-{\rm{Lmin}})/({\rm{Lmax}}+{\rm{Lmin}}),$$

where Lmax and Lmin are the luminances of the bright and dark bars respectively. The contrast of the Landolt C optotypes was defined as Weber contrast (C_W_):

$${{\rm{C}}}_{{\rm{W}}}=({{\rm{L}}}_{{\rm{O}}}-{{\rm{L}}}_{{\rm{B}}})/({{\rm{L}}}_{{\rm{B}}}),$$

where L_O_ is the luminance of the optotype and L_B_ is the luminance of the immediately adjacent background. For all participants, the experiments were completed in the order listed below.

#### Spectral sensitivity

To measure spectral sensitivity, the dLCD was incorporated into a Maxwellian viewing system, giving an 8° diameter circular stimulus display. The light source was a diffraction grating monochromator (with effective output of 420–800 nm and 10 nm full width at half maximum bandwidth; model K3031505 from Cecil Instruments Ltd, Cambridge, UK) with white LED primary illumination. A variable neutral density filter placed between the primary source and spectrometer input window allowed adjustment of spectrometer output luminous intensity. Wavelength and intensity of the monochromator output were determined for each test wavelength using a solid-state spectrometer (from Ocean Optics Ltd, USA, model HR2000+), which was also used to confirm equiluminance for representative test greyscales at each wavelength increment. Monochromator output luminous intensity was adjusted to be constant for all test wavelengths (200 photon counts per ms; 10ms integration time) by adjusting the variable density input filter.

Monocular adaptive contrast thresholds for discriminating the orientation of a square-wave grating were measured for test wavelengths varying in 10 nm increments from 435 nm to 535 nm. A grating of 5 cycles per degree (cpd) was used because: (i) this periodicity is near the optimum for perception; and (ii) it ensured that a sufficient number of cycles were present within the 1 degree viewing aperture required for the spatial distribution experiment described below. Grating orientation was randomised between trials in one of four orientations (horizontal, vertical, right oblique or left oblique), and each datum was calculated from the mean of three runs (3 × 24 trials in total). In two participants, additional measures were completed for test wavelengths between 550 and 800 nm, using 25 nm increments.

#### Spatial sensitivity and resolution to polarized light

To determine the spatial sensitivity and resolution of human polarization perception, the dLCD was mounted on the optical bench at a viewing distance of 50 cms. Waveband output of the dLCD was modified by inserting a blue filter (Lee filter # 075, ‘evening blue’; peak transmission 440–460 nm, from Lee Filters Ltd, UK) between the back face of the liquid crystal cell and the integral light source to generate a light output peaking at 460 nm. The spectral characteristics (see Supplementary Material) of the device were confirmed using a spectrometer and were not altered by the insertion of the front polarizer. The mean luminance of the display with and without the front polarizer in place was 4 cd/m^2^ and 5 cd/m^2^, respectively. The CIE coordinates of the display with or without the polarizer were x = 0.143 and y = 0.025.

To quantify the spatial distribution of sensitivity to polarized light, the front polarizer was omitted and a mask with a central 1° diameter circular aperture was placed over the face of the dLCD. Fixation points were marked on the screen in a regular 1° interval grid pattern. Participants were asked to maintain fixation at a given grid-point during each test run, up to a maximum of 5° from the test aperture. Equivalent contrast threshold of a 5 cpd grating stimulus was determined using FrACT for the right (dominant) eye of all participants.

For determining sensitivity to grating patterns, the mask had an 8° square aperture and testing was performed both with and without the front polarizer *in situ* for luminance and polarization measures respectively. Binocular contrast thresholds for discriminating the orientation of square-wave gratings were assessed in a 4-ACF paradigm using FrACT, as described above, for grating periodicities ranging from 1 to 18 cpd. The gratings were presented in one of four random orientations on each trial (horizontal, vertical, right oblique or left oblique). Using the same paradigm, but in a separate experiment, binocular polarization contrast thresholds for discriminating the orientation of Landolt C optotypes were assessed for letter sizes varying from 10 to 200 arcminutes diameter, corresponding to a visual acuity range of 0.0–1.6 logMAR. The Landolt C opening was presented in one of four random positions on each trial (up, down, right or left). For both sets of measures, each datum was determined from the mean of three runs (3 × 24 trials in total).

### Data availability

The datasets of the current study used to generate the figures are given in Supplementary Material. Complete data sets are available from the corresponding author upon request.

## Results

### Spectral sensitivity

**Figure 1:**
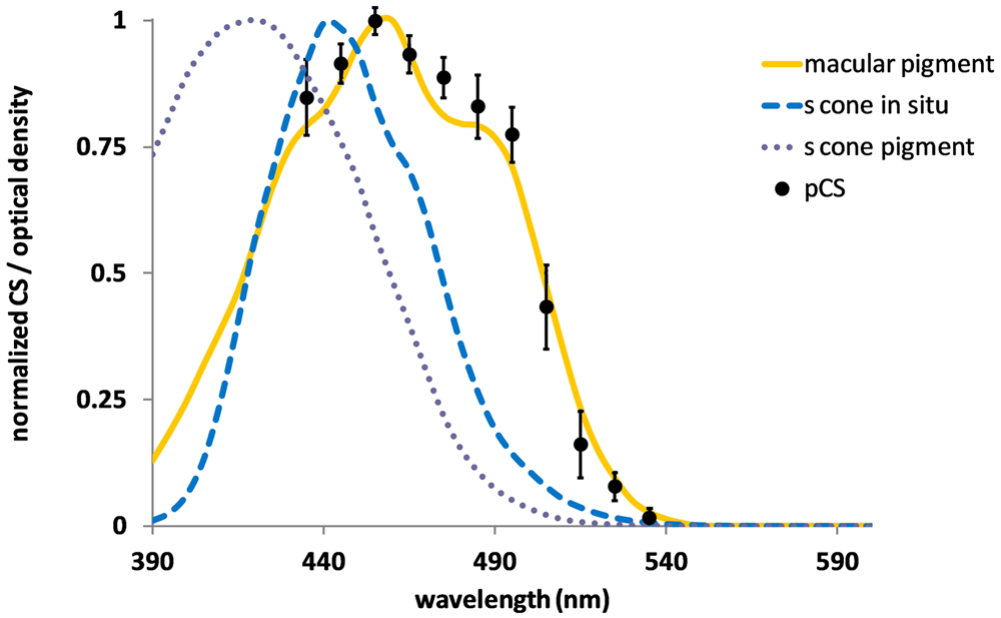
Spectral characteristics of polarization pattern perception. The solid symbols are the normalized polarization equivalent monocular contrast sensitivity measures for discriminating the orientation of a 5 cpd grating, plotted as a function of stimulus wavelength (nm). The results were averaged across five observers; the error bars show ± 1 sem. Also shown are the normalized absorption spectra of: (i) macular pigment (yellow continuous line); (ii) *in situ* s-cones (blue dash line); and (iii) s-cone photopigment (purple dot line), as published in Stockman *et al*.^23^.

The solid symbols in Fig. 1 are the normalized polarization equivalent contrast sensitivity measures for discriminating the orientation of a 5 cpd grating, plotted as a function of stimulus wavelength. The results were averaged across five observers. Note that sensitivity extended from near the lower wavelength limit of visual perception to approximately 540 nm, peaking at 460 nm. The target was not visible for test wavelengths greater than 540 nm, as confirmed with measures completed on two observers.

Also shown in Fig. 1 are the normalized absorption spectra of macular pigment, *in situ* s-cones and s-cone photopigment^23^. While there is no similarity between the polarization sensitivity measures and either the s-cone pigment or s-cone photoreceptor absorption spectra, there is a clear correspondence between our sensitivity measures and the macular pigment absorption spectrum.

### Spatial distribution of sensitivity to polarized light

**Figure 2:**
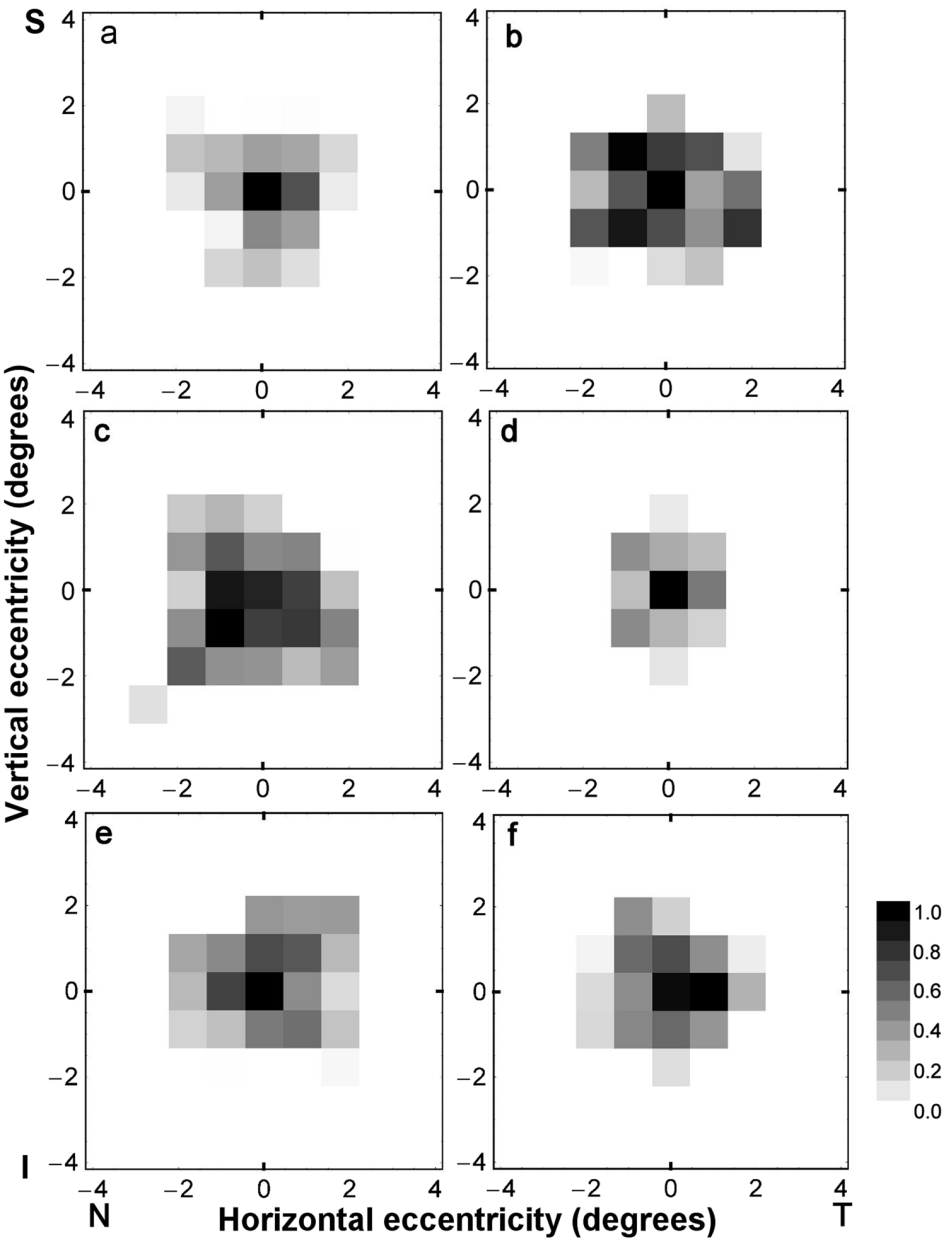
Distribution of polarization pattern perception in visual space. Two-dimension grey-scale maps for six observers (**a**–**f**) of monocular normalized log sensitivity across the central visual field for discriminating the orientation of a polarization-modulated grating of 5 cpd presented in a 1° diameter aperture. In each panel the origin of co-ordinates indicates foveal viewing and darker shades represent higher sensitivities (grey-scale range 0–1). Axes are in degrees of eccentricity from central fixation, where positive values indicate temporal (T)/superior (S) visual field and negative values indicate nasal (N)/inferior (I) visual field.

Figure 2(a–f) shows, for six observers, two-dimensional grey-scale maps of normalized log sensitivity across the central visual field for discriminating the orientation of a polarization-modulated grating of 5 cpd periodicity. In each panel, the origin of co-ordinates indicates foveal viewing and darker shades represent higher sensitivities. Note that sensitivity was highest with foveal viewing and declined sharply with retinal eccentricity at a similar rate in each quadrant of the visual field. The target stimulus was not visible at retinal eccentricities greater than 3°.

**Figure 3:**
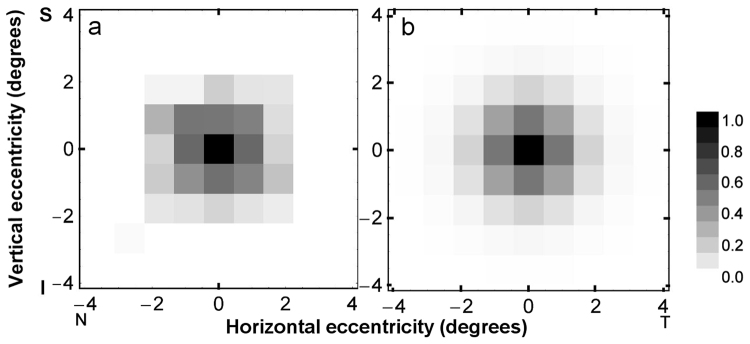
Distribution of polarization pattern perception in visual space. (a) Two-dimensional grey-scale map of the grand average of normalized log contrast sensitivity of six observers (from Fig. 2). (**b**) Two-dimensional grey-scale map of normalized typical human macular pigment density, as calculated from the model of Berendschot and van Norren^24^, where the origin indicates the fovea and darker shades indicate higher pigment density. Axes are in degrees eccentricity from central fixation, where positive values indicate temporal (T)/superior (S) visual field and negative values indicate nasal (N)/inferior (I) visual field. See Supplementary Material for details of the calculation method.

Figure 3(a) shows the distribution of polarization pattern perception averaged across the six observers (from Fig. 2). Using the same display resolution, a two-dimensional grey-scale map of typical human macular pigment density is shown in Fig. 3(b), where the origin indicates the fovea and darker shades indicate higher pigment density. It was generated by projecting into two-dimensional space a typical one-dimensional profile of macular pigment density – the parameters chosen for this projection fall within the range of density measures given by Berendschot and van Norren^24^, and are detailed together with the calculation method in the Supplementary Material. Consistent with numerous reports on the spatial distribution of macular pigment^25,26^, Fig. 3(b) shows that the concentration of pigment peaks in the centre of the macula and decreases rapidly to undetectable levels at 3° to 4° eccentricity. Note the correspondence between our quantitative data on the spatial distribution of polarization sensitivity (Fig. 3a) and a typical normalized distribution of macular pigment density (Fig. 3b).

### Spatial resolution for grating patterns and Landolt C optotypes

**Figure 4:**
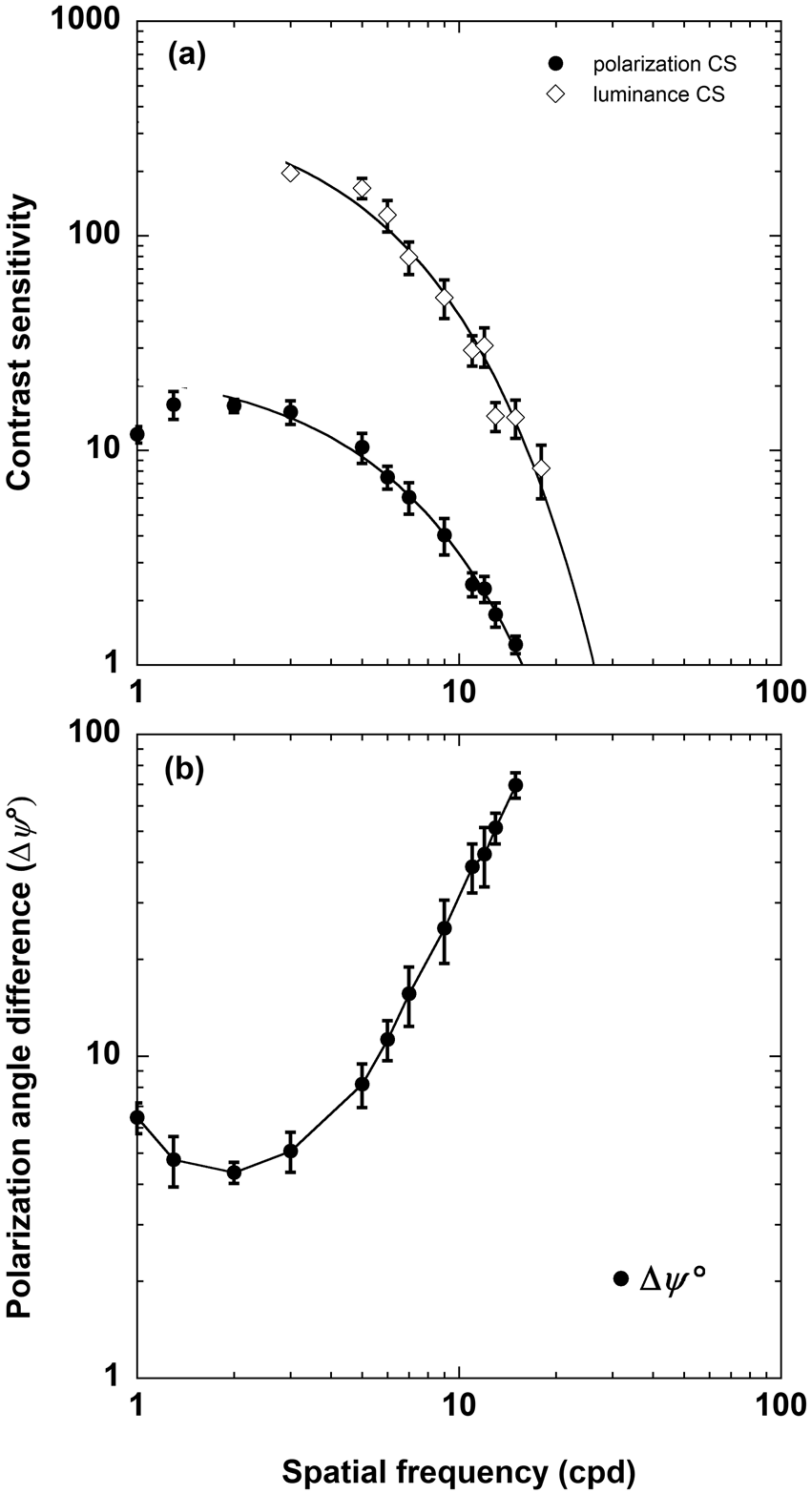
Polarization contrast sensitivity function. (**a**) Binocular contrast sensitivity measures for discriminating the orientation of polarization-modulated (solid symbols) and luminance-modulated (open symbols) square-wave gratings as a function of grating periodicity (cpd). The results were averaged across six observers. All measures were obtained using the dLCD with a blue filter (peak wavelength 460 nm) in place. The error bars show ± 1 sem. The continuous line through each data set shows an exponential function, fitted to the data points associated with the decline in sensitivity using a least-squares minimisation procedure, extrapolated to the abscissa to estimate acuity (R^2^ was 0.96 and 0.99 for the fit to luminance and polarization data, respectively). (**b**) Contrast sensitivity measures for polarization-modulated patterns replotted in terms of the minimum detectable polarization angle difference (Δ*ψ*°).

The solid symbols in Fig. 4a show the contrast sensitivity measures, averaged across six observers, for discriminating the orientation of polarization-modulated square-wave gratings as a function of grating periodicity (cpd). For comparison, sensitivity measures are also shown for luminance-modulated gratings (open symbols). All measures were obtained using a backlighting filtered to a waveband peaking at 460 nm, chosen to approximate the maximum spectral sensitivity of human polarization vision (see Supplementary Material Figure S2). Note that, over the range of values assessed, contrast sensitivity for polarization-modulated patterns is an order of magnitude less than that for luminance-modulated patterns. When plotted on logarithmic co-ordinates, an exponential function provided an excellent fit (R^2^ > 0.95) to the decline in sensitivity at higher spatial frequencies^27^ (continuous line through each data set). Extrapolating the functions to a contrast sensitivity of one (100% contrast) yields a spatial acuity of 25.8 cpd (0.07 logMAR, ~6/7.5+ Snellen equivalent) for luminance-modulated patterns and 15.4 cpd (0.29 logMAR, ~6/12+) for polarization-modulated patterns. Note that these acuities were recorded using a mean display luminance of ≤5 cd/m^2^.

In Fig. 4b the contrast sensitivity measures for polarization-modulated patterns are replotted in terms of the minimum detectable polarization angle difference (Δ*ψ*°, see Supplementary Material for details and Figure S1). The smallest angular difference observed was 4.4°, a value similar to **e**-vector angle discrimination values reported for several invertebrate species^28^.

**Figure 5:**
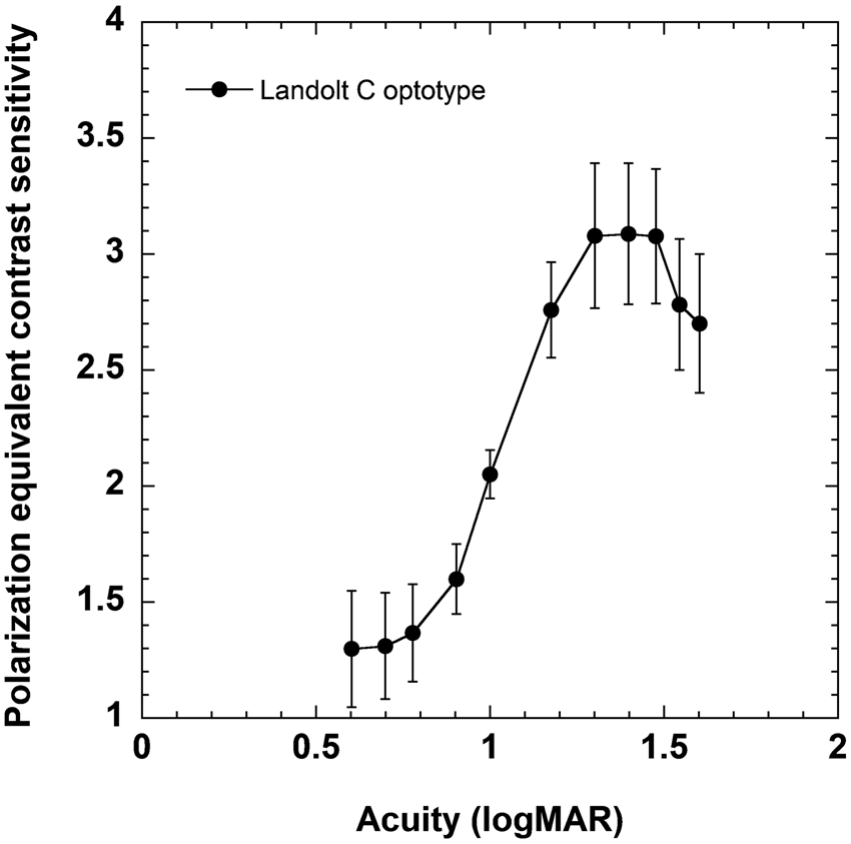
Binocular polarization equivalent contrast sensitivity for resolving Landolt C optotypes. The results were averaged across six observers. The vertical errors bars show ± 1 sem.

Contrast sensitivity, averaged across six observers, for resolving polarization-modulated Landolt C letters is shown in Fig. 5. Under the conditions of our experiment, sensitivity was maximal for letters corresponding to a logMAR acuity of approximately 1.4 (~6/150 Snellen equivalent), and declined for both smaller and larger letters. The smallest polarization-defined letter that could be resolved corresponded to an acuity of 0.6 logMAR (~6/24 Snellen equivalent).

## Discussion

In quantifying the spatial contrast sensitivity and spatial resolution of human polarization perception, we show that polarization-modulated patterns are not only identifiable across a range of contrasts and scales, but they can be resolved with an acuity approaching that for luminance-modulated patterns (Fig. 4). We also show that the perception of polarization patterns is dependent on the absorption properties and spatial extent of macular pigment (Figs 1, 2 and 3). Given these findings, we suggest that polarization-modulated stimuli may be a highly efficient and directed means of assessing central retinal (macular) function in health and disease.

### Polarization pattern perception: comparison with Haidinger’s brushes

The results of the present study make it clear that human polarization perception is not confined to the phenomenon of Haidinger’s brushes. The Haidinger effect is perhaps the simplest manifestation of human polarization sensitivity and its biophysical basis has been discussed at length elsewhere, with general agreement that the effect is due to selective absorption by oriented macular pigment molecules^6^. In Fig. 1 we show that the spectral characteristics of polarization-modulated patterns are almost identical to those detailed by Bone^19^ for Haidinger’s brushes: the spectral characteristics of both stimulus types show a clear qualitative correspondence with the absorption spectra of macular pigment. In Figs 2 and 3a we show that sensitivity to polarization-modulated patterns declines to zero at retinal eccentricities beyond 3°, which is consistent with the known areal extent of Haidinger’s brushes^7,8,20^ and matches typical values for the spatial distribution of macular pigment density (Fig. 3b). In short, the similarities reported here between polarization pattern perception and the Haidinger effect, both critically dependent on the presence of a radially diattenuating macula, support the view^14^ that they are manifestations of the same phenomenon of human polarization sensitivity.

Apart from the diattenuating properties of the macula, polarization contrast sensitivity may also be influenced by the ocular media. Light scatter from media opacities (e.g. cataracts) may depolarize the incident light, thus reducing polarization pattern perception. Ocular retardation, predominantly the result of corneal birefringence^29^, varies between individuals^30^ and may need to be considered in any quantitative interpretation of macular diattenuation. This may be particularly relevant for measuring macular pigment optical density using polarization perception^13^. In our study, however, we note there was little inter-observer variability in on-axis (central viewing) measures (Fig. 4), and conclude that, for the participants of this study, inter-individual variations in the participants’ ocular media had limited effect on polarization contrast sensitivity.

### Quantification of human polarization pattern perception

The contrast sensitivity function (CSF) for polarization-modulated gratings peaked near 2 cpd and declined to a projected minimum at 15.4 cpd (see Fig. 4a). This acuity is within a factor of two of that projected for luminance-modulated gratings, as determined under the same experimental conditions (i.e. a blue display screen of mean luminance ≤ 5 cd/m^2^).

**Figure 6:**
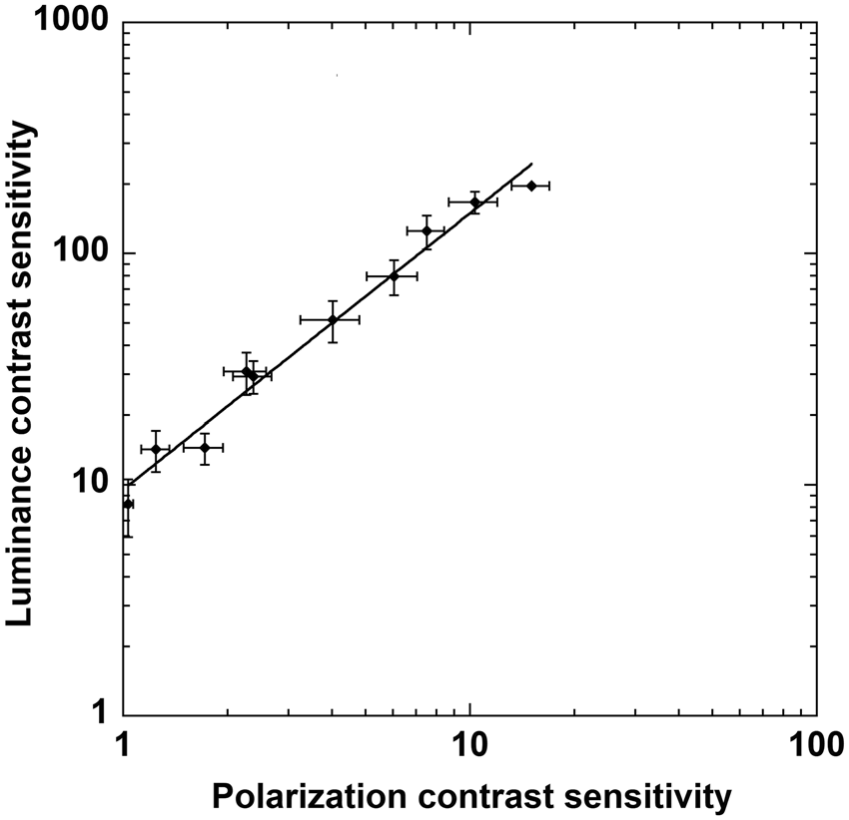
Relationship between polarization contrast sensitivity (x-axis, pCS) and luminance contrast sensitivity (y-axis, LCS). The solid line is the geometric regression equation LCS = 9.56 x pCS^1.19^ (R = 0.98). Error bars show ± 1 sem.

The decline in sensitivity for polarization-modulated gratings was qualitatively, though not quantitatively, similar to that measured for luminance-modulated gratings (compare open and closed symbols in Fig. 4a), which is to be expected if both stimulus types were detected via a common neural mechanism. In support of this we note that the CSF for polarization-modulated gratings is similar in peak frequency and general shape to previously measured CSFs for luminance-modulated gratings illuminated by blue light-emitting diodes (see Fig. 6 in Ramamurthy *et al*.^31^). The results presented in Fig. 4a can be interpreted in terms of the macular response: the luminance signal needs to be approximately one order of magnitude less than the polarization signal to generate the same macular response. This relationship between polarization and luminance contrast sensitivities (pCS, LCS) is shown in Fig. 6, where the data fits the geometric expression LCS = 9.56 x pCS^1.19^ (R = 0.98).

Together with the spectral characteristics and sensitivity profile of polarization pattern perception, these findings provide further support for the radial diattenuator model of polarization sensitivity. This model posits that radially orientated diattenuating macular structures convert orientation of linear polarization into a luminance contrast signal, which can then be processed in normal fashion without recourse to any specialised polarization-sensitive ocular or neural mechanisms.

Our sensitivity measures for resolving Landolt-C letters (Fig. 5) show that polarization-modulated stimuli can be adapted to standard optotypes as used in clinical practice. For the viewing conditions employed, sensitivity peaked at letter sizes corresponding to 1.4 logMAR and declined for both smaller and larger letters. The decline in sensitivity for smaller letters is consistent with the resolution properties of the eye^27^. The decline in sensitivity with the three largest Landolt C optotypes used here (diameter 150–200 arcminutes) may reflect the fact that their openings were imaged on paracentral regions of the macula where human polarization sensitivity is rapidly declining (see Fig. 3).

### Comparison with polarization sensitivity in animals

Using the relationship shown in Supplementary Material Figure S1a, the polarization equivalent contrast sensitivity measures were replotted in terms of the minimum polarization angle difference that was detectable between the foreground and background of our grating stimuli. The results, shown in Fig. 4b, indicate that humans are capable of detecting an edge between adjacent polarized areas that differ in linear polarization by as little as 4.4° (achieved with grating periodicities of 2 cpd). These findings, which are without precedent for terrestrial vertebrates, compliment those of Temple *et al*.^13^ who demonstrated that humans are sensitive to partially polarized light. The ability to discriminate angular differences in **e**-vector in the range of 10°-20° is reported in invertebrates and aquatic vertebrates^32–34^ and rises to 3.2° for fiddler crabs^35^. The most acute **e**-vector angle discrimination reported for any animal (Mourning Cuttlefish) is just over 1°, a resolution thought to be high enough to provide functional advantages in real-world visual tasks, including object recognition^28^.

The minimum polarization angle difference we report here (Fig. 4b) may underestimate the most acute **e**-vector angle discrimination possible in human vision. This is so because the polarization output from the dLCD for low contrasts was to some extent elliptically polarized (maximum *χ* = 11°) and depolarized (minimum DOP = 90%), which would give rise to lower luminous intensities at the photoreceptor level than if the stimulus was purely linearly polarized (see Supplementary Material).

Despite this high sensitivity to polarization orientation, however, it remains doubtful that such information yields any adaptive advantage for present-day humans. A key requirement to successfully navigate our natural world is the ability to detect lines and edge boundaries, and neural mechanisms highly sensitive to luminance and chromatic contrast have evolved within human vision for just this purpose (see e.g. Mullen *et al*.^36^). We show here that, across a wide range of spatial scales, contrast sensitivity for detecting luminance-modulated targets is an order of magnitude greater than that for detecting polarization-modulated targets (Figs 4a and 6). Given this, it seems reasonable to suppose that edge boundaries and other significant visual features within our natural environment would be more readily detected through luminance and/or chromatic signals than polarization signals. In the absence of any alternative evidence, we assume that human polarization sensitivity is an epiphenomenon resulting from the fortuitous coexistence of macular radial symmetry and a mechanism of diattenuation incorporating macular pigment.

### Clinical significance of polarization pattern perception

Although polarization perception has doubtful functional significance for humans in real-world settings, its dependence on macular pigment and normal macular spatial ordering suggests that measures of contrast sensitivity using polarization-modulated targets, including optotypes, may provide a unique means of quantifying any disruption in normal macular structure or function. While the clinical potential of Haidinger’s brushes has been extensively documented^10,13,37–39^, this approach does not enable the quantification of polarization sensitivity across a broad range of spatial scales and contrasts. The latter may be particular useful in early identification of macular dysfunction due to age-related macular degeneration, a disease which remains the commonest cause of blindness in the developed world but one that, if diagnosed early, is potentially receptive to treatments including dietary supplementation^40^ and direct therapeutic interventions^41^. Finally, the close association between the absorption spectrum of macular pigment and wavelength dependence of polarization contrast sensitivity (Fig. 1) suggests a potential use of this metric in determining macular pigment density, a physiological parameter known to correlate with the susceptibility of developing macular disease^42^.

## Electronic supplementary material

Supplementary Material
