# Supplementary material for: The spectral, spatial and contrast sensitivity of human polarization pattern perception
Source: Sci Rep. 2017 Nov 29;7:16571. doi: 10.1038/s41598-017-16873-6 (PMC5707437; doi:10.1038/s41598-017-16873-6)
Supplement: Supplementary file 1 — Supplementary Material [file 41598_2017_16873_MOESM1_ESM.pdf]

Supplementary Material

The spectral, spatial and contrast sensitivity of human polarization pattern perception

Gary P. Misson, Stephen J. Anderson

Polarized light and polarimetry. If light is considered to be an electromagnetic wave then polarization is the property of the electric field vector (**e**-vector) to behave in a defined way. The **e**-vector is described by two hypothetical orthogonal components in a plane centred on and perpendicular to the direction of propagation. A general description of polarized light (elliptical polarization) results from the two **e**-vector components oscillating with a phase difference between them. The resultant **e**-vector rotates with time to describe an ellipse with an ellipticity and orientation that is dependent on the phase difference and magnitude of its components. The **e**-vector rotates clockwise (right-handed) or anticlockwise (left-handed), depending on the relative retardation of the two components. There are two specific cases: (i) In linear polarization the **e**-vector components are in phase (or one component has zero magnitude), confining the **e**-vector to vibrate in a given plane along the direction of propagation – the orientation of the **e**-vector plane is determined by the relative magnitude of its components; and (ii) In circular polarization the components have equal magnitude and a phase difference of one quarter wavelength – the **e**-vector then rotates in a right- or left-handed circle about the direction of propagation. Thus polarization can be defined by orientation of the ellipse/plane of polarization (polarization orientation, $0 \leq \psi \leq \pi$), the degree to which it is elliptical (ellipticity angle, $\chi \leq \pm \pi/4$) with plane (linear, $\chi = 0$) and circular ($\chi = \pm \pi/4$) polarization as extreme cases, and handedness (right, $\chi > 0$; left, $\chi < 0$) for elliptical and circular polarization. A further parameter, the degree of polarization (DOP, percentage polarization) is the extent to which a beam of light is polarized.

The polarization output of the dLCD was determined using an optical bench-mounted polarimeter comprising a Fresnel rhomb achromatic quarter-wave

retarder, a Glan-Thompson polarizer and an Ocean Optics spectrometer with appropriate software. The method is described elsewhere [1,2] and determines polarization angle, ellipticity and DOP.

The relationship shown in Fig. S1a between greyscale (gr) and polarization angle in degrees ($\psi°$) is described by the second order polynomial,

$\psi° = 0.0009gr^2 + 0.1087gr + 46.865$

For FrACT, and for the dLCD used in this study, the relationship between Michelson contrast (C_M) and the respective greyscales/polarization angles ($\psi°$) is given in Fig. S1b. This figure also shows the near linear relationship between the difference in foreground/background polarization angle ($\Delta\psi°$) of a figure and its Michelson contrast (C_M), described by:

$\Delta\psi° = 0.8499\ C_M - 1.0055$

For example, an image with maximum luminance contrast of 100% when viewed with the front polarizer *in situ* is generated by an image with foreground and background greyscale values of 000 and 255, respectively. The polarization output of the same image with the front polarizer removed (i.e. with equivalent contrast $_{PEQ}C$ = 100%) will be ψ = 43° for the foreground and ψ = 130° for the background, with a polarization angle difference of $\Delta\psi$ = 87° and polarization angle contrast of $_{P}C$ = 97%. Note that a polarization contrast of 100% cannot be achieved because of the limitations of LCD technology.

Similar relationships exist for χ and DOP. The present study determines the perceptibility of linear polarized light. Polarimetry is therefore used to determine if χ and DOP are confounding variables in the data. Both χ and DOP have a non-linear relationship with greyscale and hence contrast. DOP reaches a minimum value of 0.90 at a greyscale value of 174 and χ has a maximum of 11° at a greyscale value of 131. For greyscales of 0 and 255, DOP = 1.0 and 0.95, respectively, and $\chi \leq 2°$ and 0, respectively. These departures from pure polarization and linearity are unlikely to

significantly affect the results of the study, but if so will lead to an underestimate of resolution and contrast sensitivity.

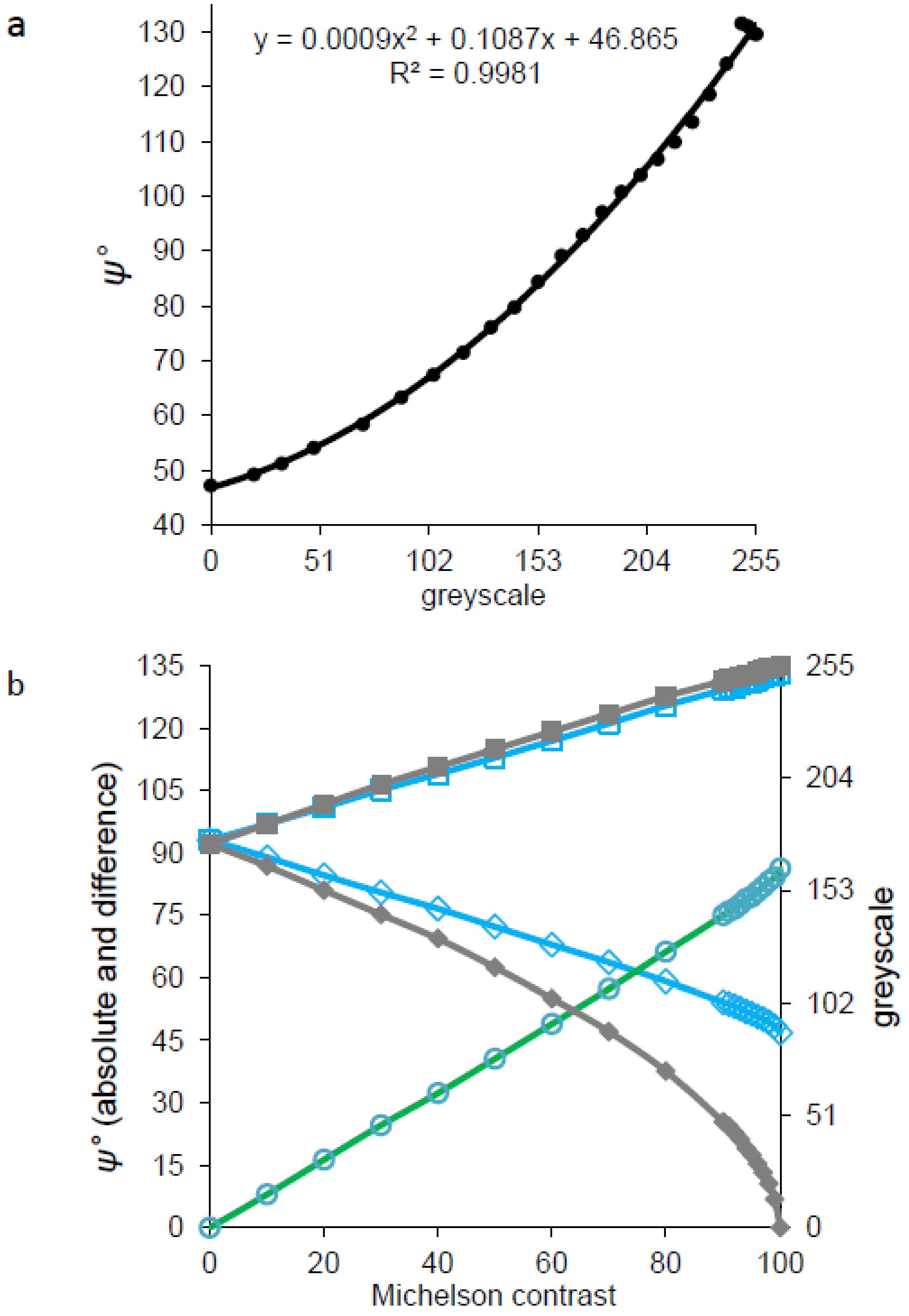

Figure S1. (a) Relationship between the greyscale values of the delaminated liquid crystal display (dLCD) and polarization angle (ψ°). (b, left vertical axis) The blue lines with open symbols show the relationship between Michelson contrast (C_M) and polarization angle (ψ°). The difference in foreground/background polarization angles ($\Delta\psi$°, left vertical axis) is shown in green. (b, right vertical

axis). The grey lines with filled symbols show the relationship between C_M and the greyscale values of the dLCD. See text for explanation.

Projection of macular pigment optical density (MPOD) to 2-d space. The one-dimensional profiles of various MPOD patterns are modelled by Berendschot and van Norren [3] according to:

$$\text{MPOD(x)} = A_1 10^{-\rho_1 x} + A_2 10^{-\rho_2 (x - x_2)^2} \qquad \text{Eq. 1}$$

with variables and parameters as defined in table S1. This can be extended to 2-d Cartesian (*x, y*) space and a *y*-scaling (ellipticity) factor, *f*, introduced thus:

$$\text{MPOD}(x, y, f) = A_1 10^{-\rho_1 \sqrt{x^2 + (y/f)^2}} + A_2 10^{-\rho_2 (\sqrt{x^2 + (y/f)^2} - x_2)^2} \qquad \text{Eq. 2}$$

Equation 2 was used to plot a theoretical distribution of MPOD in Cartesian coordinates (Main Text Figure 3b).

Table S1 Parameters and variables from Berendschot and van Norren[3]

	Parameter	Value range	Value used in present study
A_1	amplitudes of the distributions	0.28 ± 0.13 reflectance<br>0.31 ± 0.12 autofluorescence	0.3
A_2		0.13 ± 0.07<br>0.11 ± 0.08	0.12
ρ_1	peakedness	$0.38 \pm 0.24°$	0.5
ρ_2		$1.2 \pm 1.1\ \text{deg}^2$	0.3
x_2	eccentricity at which the Gaussian distribution peaks	$0.70 \pm 0.66°$	0.7
f	ellipticity (y-axis scaling)	1 = circular<br><1 = prolate ellipse (scales y up)<br>>1 = oblate ellipse (scales y down)	1

Spectral characteristics of dLCD light source. The output spectrum of the dLCD with and without the blue filter in place is shown in Figure S2. The MPOD function is shown for comparison. Note that the dLCD output approximates to the macular pigment absorption peak.

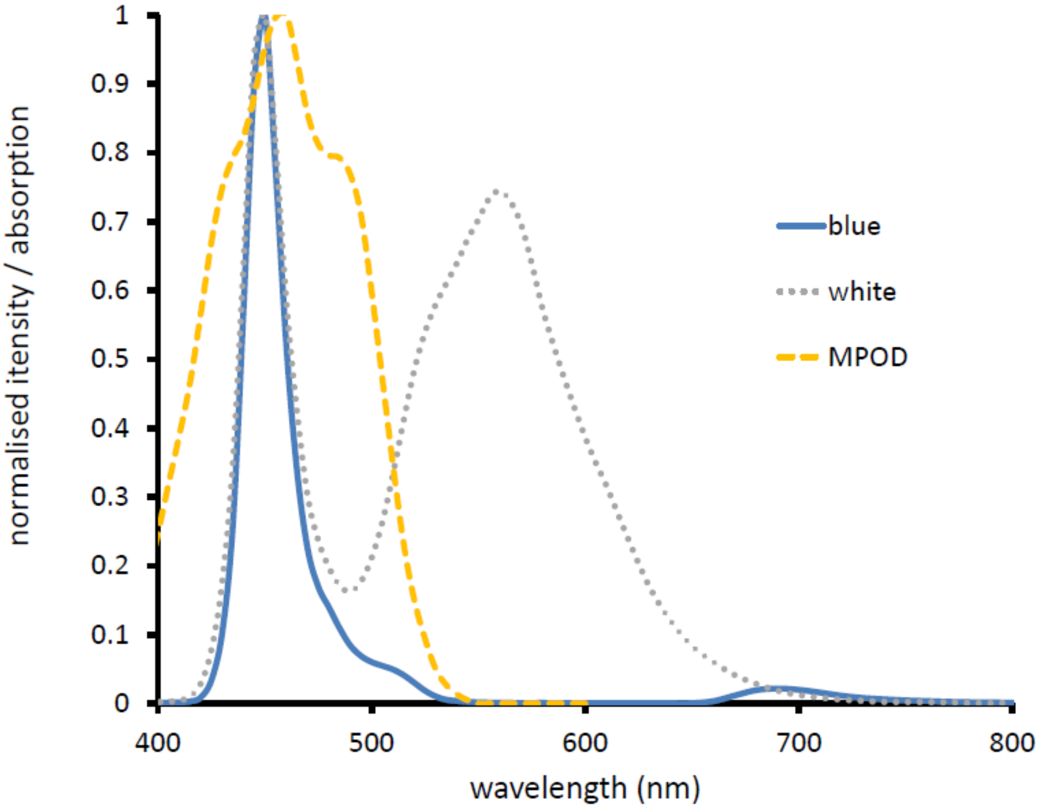

Figure S2**.** Spectral output characteristics of the delaminated liquid crystal display (dLCD) with either the LCD white light source alone (broken grey line) or with a blue filter in place (solid blue line). For comparison, the macular pigment optical density (MPOD) function is also shown (broken yellow line).

Supplementary Material References

1 Temple, S. E. *et al.* Perceiving polarization with the naked eye: characterization of human polarization sensitivity. *Proc. R. Soc. B* **282**, 20150338, doi:10.1098/rspb.2015.0338 (2015).

2 Born, M. & Wolf, E. *Principles of Optics*. 7 edn, (Cambridge University Press, 2005).

3 Berendschot, T. T. & van Norren, D. Macular pigment shows ringlike structures. *Invest Ophthalmol Vis Sci* **47**, 709-714, doi:10.1167/iovs.05-0663 (2006).

Data for figures

Figure 1

Spectral characteristics of MP, cones and pCS

wlth	MPOD	s cone	s cone	pCS norm		
(nm)	mac pgt	in situ	pigment	mean	sem	dLCD01
390	0.1294	0.0108	0.7347			0.0007
395	0.1854	0.0266	0.8113			0.0007
400	0.2480	0.0626	0.8915			0.0010
405	0.3200	0.1336	0.9423			0.0014
410	0.3900	0.2511	0.9788			0.0017
415	0.4660	0.4060	0.9945			0.0031
420	0.5660	0.5719	1.0000			0.0089
425	0.6700	0.7011	0.9874			0.0338
430	0.7480	0.8245	0.9502			0.1021
435	0.7920	0.9177	0.8913	0.8481	0.0749	0.2587
440	0.8240	0.9949	0.8297			0.5717
445	0.8800	0.9842	0.7584	0.9157	0.0385	0.8986
450	0.9520	0.9380	0.6812			1.0000
455	0.9960	0.8352	0.5757	1.0000	0.0275	0.7779
460	1.0000	0.7556	0.4846			0.5230
465	0.9340	0.7015	0.3970	0.9336	0.0367	0.3399
470	0.8560	0.6076	0.3042			0.2252
475	0.8120	0.4803	0.2174	0.8882	0.0394	0.1698
480	0.7960	0.3593	0.1525			0.1396
485	0.7920	0.2644	0.1076	0.8312	0.0627	0.1095
490	0.7680	0.1910	0.0738			0.0843
495	0.7100	0.1433	0.0514	0.7756	0.0549	0.0691
500	0.5980	0.1085	0.0348			0.0602
505	0.4720	0.0778	0.0220	0.4347	0.0829	0.0547
510	0.3460	0.0527	0.0132			0.0494
515	0.2320	0.0367	0.0082	0.1627	0.0656	0.0412
520	0.1500	0.0248	0.0051			0.0300
525	0.0940	0.0163	0.0032	0.0795	0.0279	0.0186
530	0.0500	0.0105	0.0019			0.0104
535	0.0266	0.0067	0.0012	0.0173	0.0166	0.0056
540	0.0131	0.0042	0.0007			0.0037
545	0.0049	0.0026	0.0004			0.0025
550	0.0000	0.0016	0.0003			0.0017
555	0.0000	0.0010	0.0002			0.0014
560	0.0000	0.0006	0.0001			0.0010
565	0.0000	0.0004	0.0001			0.0010
570	0.0000	0.0002	0.0000			0.0006
575	0.0000	0.0001	0.0000			0.0007
580	0.0000	0.0001	0.0000			0.0010
585	0.0000	0.0001	0.0000			0.0004
590	0.0000	0.0000	0.0000			0.0008
595	0.0000	0.0000	0.0000			0.0005
600	0.0000	0.0000	0.0000			0.0006
605	0.0000	0.0000	0.0000			0.0008

Figure 2. Subject Data for Density plots

Normalized logCS

Subject a	-4	-3	-2	-1	0	1	2	3	4
4	0.000	0.000	0.000	0.000	0.000	0.000	0.000	0.000	0.000
3	0.000	0.000	0.000	0.000	0.000	0.000	0.000	0.000	0.000
2	0.000	0.000	0.000	0.165	0.244	0.132	0.000	0.000	0.000
1	0.000	0.000	0.000	0.044	0.468	0.378	0.000	0.000	0.000
0	0.000	0.000	0.087	0.382	1.000	0.683	0.079	0.000	0.000
-1	0.000	0.000	0.236	0.279	0.378	0.348	0.156	0.000	0.000
-2	0.000	0.000	0.044	0.000	0.005	0.009	0.000	0.000	0.000
-3	0.000	0.000	0.000	0.000	0.000	0.000	0.000	0.000	0.000
-4	0.000	0.000	0.000	0.000	0.000	0.000	0.000	0.000	0.000

Subject b	-4	-3	-2	-1	0	1	2	3	4
4	0.000	0.000	0.000	0.000	0.000	0.000	0.000	0.000	0.000
3	0.000	0.000	0.000	0.000	0.000	0.000	0.000	0.000	0.000
2	0.000	0.000	0.031	0.000	0.139	0.241	0.000	0.000	0.000
1	0.000	0.000	0.667	0.910	0.699	0.438	0.802	0.000	0.000
0	0.000	0.000	0.273	0.663	1.000	0.372	0.558	0.000	0.000
-1	0.000	0.000	0.503	0.989	0.768	0.687	0.108	0.000	0.000
-2	0.000	0.000	0.000	0.000	0.258	0.000	0.000	0.000	0.000
-3	0.000	0.000	0.000	0.000	0.000	0.000	0.000	0.000	0.000
-4	0.000	0.000	0.000	0.000	0.000	0.000	0.000	0.000	0.000

Subject c	-4	-3	-2	-1	0	1	2	3	4
4	0.000	0.000	0.000	0.000	0.000	0.000	0.000	0.000	0.000
3	0.000	0.124	0.000	0.000	0.000	0.000	0.000	0.000	0.000
2	0.000	0.000	0.644	0.434	0.423	0.267	0.384	0.000	0.000
1	0.000	0.000	0.445	1.000	0.757	0.781	0.488	0.000	0.000
0	0.000	0.000	0.186	0.913	0.861	0.753	0.243	0.000	0.000
-1	0.000	0.000	0.413	0.657	0.462	0.482	0.004	0.000	0.000
-2	0.000	0.000	0.211	0.288	0.183	0.000	0.000	0.000	0.000
-3	0.000	0.000	0.000	0.000	0.000	0.000	0.000	0.000	0.000
-4	0.000	0.000	0.000	0.000	0.000	0.000	0.000	0.000	0.000

Subject d	-4	-3	-2	-1	0	1	2	3	4
4	0.000	0.000	0.000	0.000	0.000	0.000	0.000	0.000	0.000
3	0.000	0.000	0.000	0.000	0.000	0.000	0.000	0.000	0.000
2	0.000	0.000	0.001	0.000	0.104	0.000	0.001	0.000	0.000
1	0.000	0.000	0.001	0.460	0.295	0.177	0.001	0.000	0.000
0	0.000	0.000	0.001	0.255	1.000	0.524	0.001	0.000	0.000
-1	0.000	0.000	0.001	0.442	0.329	0.259	0.001	0.000	0.000
-2	0.000	0.000	0.001	0.000	0.086	0.000	0.001	0.000	0.000
-3	0.000	0.000	0.000	0.000	0.000	0.000	0.000	0.000	0.000
-4	0.000	0.000	0.000	0.000	0.000	0.000	0.000	0.000	0.000

Subject e	-4	-3	-2	-1	0	1	2	3	4
4	0.000	0.000	0.000	0.000	0.000	0.000	0.000	0.000	0.000
3	0.000	0.000	0.000	0.000	0.000	0.000	0.000	0.000	0.000

2	0.000	0.000	0.000	0.006	0.000	0.000	0.035	0.000	0.000
1	0.000	0.000	0.176	0.247	0.523	0.563	0.235	0.000	0.000
0	0.000	0.000	0.274	0.740	1.000	0.453	0.144	0.000	0.000
-1	0.000	0.000	0.350	0.462	0.703	0.654	0.275	0.000	0.000
-2	0.000	0.000	0.000	0.000	0.411	0.394	0.395	0.000	0.000
-3	0.000	0.000	0.000	0.000	0.000	0.000	0.000	0.000	0.000
-4	0.000	0.000	0.000	0.000	0.000	0.000	0.000	0.000	0.000

Subject f	-4	-3	-2	-1	0	1	2	3	4
4	0.000	0.000	0.000	0.000	0.000	0.000	0.000	0.000	0.000
3	0.000	0.000	0.000	0.000	0.000	0.000	0.000	0.000	0.000
2	0.000	0.000	0.000	0.001	0.133	0.000	0.000	0.000	0.000
1	0.000	0.000	0.156	0.469	0.589	0.416	0.001	0.000	0.000
0	0.000	0.000	0.147	0.446	0.956	1.000	0.302	0.000	0.000
-1	0.000	0.000	0.049	0.582	0.696	0.444	0.074	0.000	0.000
-2	0.000	0.000	0.000	0.444	0.187	0.001	0.000	0.000	0.000
-3	0.000	0.000	0.000	0.000	0.000	0.000	0.000	0.000	0.000
-4	0.000	0.000	0.000	0.000	0.000	0.000	0.000	0.000	0.000

Figure 3: Field data. Grand Mean and MPOD simulation

Grand Average Subjects a, b, c, d, e, f

	-4	-3	-2	-1	0	1	2	3	4
4	0.000	0.000	0.000	0.000	0.000	0.000	0.000	0.000	0.000
3	0.000	0.015	0.000	0.000	0.000	0.000	0.000	0.000	0.000
2	0.000	0.000	0.067	0.078	0.198	0.074	0.051	0.000	0.000
1	0.000	0.000	0.169	0.463	0.542	0.399	0.160	0.000	0.000
0	0.000	0.000	0.147	0.582	1.000	0.539	0.175	0.000	0.000
-1	0.000	0.000	0.254	0.594	0.570	0.477	0.107	0.000	0.000
-2	0.000	0.000	0.100	0.105	0.206	0.119	0.071	0.000	0.000
-3	0.000	0.000	0.000	0.000	0.000	0.000	0.000	0.000	0.000
-4	0.000	0.000	0.000	0.000	0.000	0.000	0.000	0.000	0.000

Normalized simulated MPOD

	-4	-3	-2	-1	0	1	2	3	4
4	0.001	0.002	0.005	0.007	0.008	0.007	0.005	0.002	0.001
3	0.002	0.006	0.013	0.025	0.033	0.025	0.013	0.006	0.002
2	0.005	0.013	0.044	0.12	0.175	0.12	0.044	0.013	0.005
1	0.007	0.025	0.12	0.372	0.539	0.372	0.12	0.025	0.007
0	0.008	0.033	0.175	0.539	1	0.539	0.175	0.033	0.008
-1	0.007	0.025	0.12	0.372	0.539	0.372	0.12	0.025	0.007
-2	0.005	0.013	0.044	0.12	0.175	0.12	0.044	0.013	0.005
-3	0.002	0.006	0.013	0.025	0.033	0.025	0.013	0.006	0.002
-4	0.001	0.002	0.005	0.007	0.008	0.007	0.005	0.002	0.001

Figure 4 and Figure 6

Polarization contrast sensitivity functions: Grating stimulus

1 Contrast

	Six Subjects				
	Grating	Blue field			
logMAR	CPD	pCS	SEM	LCS	SEM
1.477121	1	11.87574	1.041211		
1.363178	1.3	16.34896	2.397585		
1.176091	2	16.12943	1.120773		
1	3	15.08046	1.88866	196.0784	0
0.778151	5	10.35151	1.655846	166.8175	17.99619
0.69897	6	7.51025	0.922016	124.8526	20.86399
0.632023	7	6.043335	1.006173	79.67707	13.67702
0.522879	9	4.024193	0.773998	51.60176	10.58519
0.435729	11	2.377387	0.303206	29.38691	4.777256
0.39794	12	2.268008	0.315639	30.80288	6.37596
0.363178	13	1.719703	0.222615	14.46083	2.236001
0.30103	15	1.244874	0.115053	14.24627	2.879002
0.221849	18	1.034819	0.034819	8.242664	2.312488

2 Delta Psi

Grating		delta psi	
logMAR	CPD	Δ psi	SEM
1.477121	1	6.450956	0.704928
1.363178	1.3	4.775945	0.85266
1.176091	2	4.354819	0.328823
1	3	5.079784	0.727183
0.778151	5	8.193251	1.273183
0.69897	6	11.28146	1.612096
0.632023	7	15.63129	3.261475
0.522879	9	24.9894	5.569676
0.435729	11	38.86874	6.761217
0.39794	12	42.43832	8.91555
0.363178	13	51.2523	5.620451
0.30103	15	69.60759	6.357076
0.221849	18	80.52116	3.463342

Figure 5

Polarization contrast sensitivity functions: Landolt C

Six subjects as for grating

Blue field			Weber Contrast Threshold				Weber Contrast Sensitivity				
MAR	logMAR	size amin	pCT	SEM	LCT	SEM	LogMAR	pCS C	sem	LCS C	sem
40.00	1.60	200.00	0.41	0.06	0.01	0.76	1.60	2.70	0.30	151.60	38.61
35.00	1.54	175.00	0.42	0.05	0.01	0.79	1.54	2.78	0.28	126.34	37.64
30.00	1.48	150.00	0.47	0.05	0.01	0.79	1.48	3.08	0.29	122.81	38.68
25.00	1.40	125.00	0.48	0.05	0.01	0.78	1.40	3.09	0.30	120.25	40.66
20.00	1.30	100.00	0.48	0.04	0.01	0.84	1.30	3.08	0.31	82.72	20.10
15.00	1.18	75.00	0.43	0.04	0.02	0.83	1.18	2.76	0.21	71.68	16.44
10.00	1.00	50.00	0.30	0.03	0.03	0.89	1.00	2.05	0.10	42.29	5.27
8.00	0.90	40.00	0.18	0.04	0.03	0.87	0.90	1.60	0.15	40.35	5.05
6.00	0.78	30.00	0.10	0.05	0.04	0.85	0.78	1.37	0.21	26.84	4.17
5.00	0.70	25.00	0.05	0.04	0.04	0.86	0.70	1.31	0.23	26.32	4.03
4.00	0.60	20.00	0.05	0.05	0.06	0.85	0.60	1.30	0.25	18.03	2.93
3.00	0.48	15.00	0.02	0.02	0.13	0.82	0.48	1.19			
2.00	0.30	10.00	0.01	0.01	0.24	0.83	0.30	1.06			

Figure S1

Angle of linear polarization (psi) for given greyscales

Grey values and psi for given contrasts

Fig S1a	Michelson	grey		psi		
	contrast %	grey fore	grey back	psi fore	psi back	delta psi
	0	174	174	93.0	93.0	0.0
	10	164	183	88.9	96.9	8.0
	20	153	192	84.6	100.9	16.3
	30	142	201	80.4	105.1	24.6
	40	131	209	76.5	108.9	32.3
	50	118	217	72.2	112.8	40.6
	60	104	225	67.9	116.9	49.0
	70	89	233	63.7	121.1	57.4
	80	71	241	59.1	125.3	66.2
	90	48	248	54.2	129.2	75.0
	91	46	249	53.8	129.7	76.0
	92	43	249	53.2	129.7	76.5
	93	40	250	52.7	130.3	77.6
	94	36	251	51.9	130.8	78.9
	95	33	251	51.4	130.8	79.4
	96	29	252	50.8	131.4	80.6
	97	25	253	50.1	132.0	81.8
	98	20	254	49.4	132.5	83.1
	99	13	254	48.4	132.5	84.1
	100	0	255	46.9	133.1	86.2

Psi for given greyscale

Fig S1b	grey	psi av
	255	129.6
	254	129.7
	251	131.1
	248	131.6
	241	124.2
	233	118.6
	225	113.6
	217	109.9
	209	106.8
	201	103.9
	192	100.8
	183	97.1
	174	92.9
	164	89.2
	153	84.4
	142	79.8
	131	76.2
	118	71.6
	104	67.5
	89	63.4
	71	58.4
	48	54.1
	33	51.3
	20	49.3
	0	47.3

Figure S2

Spectral Characteristics of blue screen, white screen and Mac Pigment

Wlth	blue	white	MPOD	Wlth	blue	white	MPOD	Wlth	blue	white	MPOD
390	0.0007	0.0019	0.1294	500	0.0602	0.2114	0.5980	610	0.0008	0.3156	*
395	0.0007	0.0024	0.1854	505	0.0547	0.2633	0.4720	620	0.0005	0.2396	*
400	0.0010	0.0019	0.2480	510	0.0494	0.3331	0.3460	630	0.0005	0.1698	*
405	0.0014	0.0027	0.3200	515	0.0412	0.4112	0.2320	640	0.0005	0.1199	*
410	0.0017	0.0042	0.3900	520	0.0300	0.4845	0.1500	650	0.0006	0.0836	*
415	0.0031	0.0104	0.4660	525	0.0186	0.5392	0.0940	660	0.0035	0.0603	*
420	0.0089	0.0290	0.5660	530	0.0104	0.5793	0.0500	670	0.0116	0.0414	*
425	0.0338	0.0783	0.6700	535	0.0056	0.6100	0.0266	680	0.0193	0.0286	*
430	0.1021	0.1754	0.7480	540	0.0037	0.6400	0.0131	690	0.0217	0.0185	*
435	0.2587	0.3479	0.7920	545	0.0025	0.6746	0.0049	700	0.0199	0.0123	*
440	0.5717	0.6376	0.8240	550	0.0017	0.7029	0.0000	710	0.0159	0.0085	*
445	0.8986	0.9416	0.8800	555	0.0014	0.7331	0.0000	720	0.0114	0.0059	*
450	1.0000	1.0000	0.9520	560	0.0010	0.7441	0.0000	730	0.0081	0.0039	*
455	0.7779	0.8134	0.9960	565	0.0010	0.7286	0.0000	740	0.0062	0.0024	*
460	0.5230	0.5872	1.0000	570	0.0006	0.6956	0.0000	750	0.0046	0.0023	*
465	0.3399	0.4255	0.9340	575	0.0007	0.6383	0.0000	760	0.0036	0.0020	*
470	0.2252	0.3023	0.8560	580	0.0010	0.5736	0.0000	770	0.0025	0.0017	*
475	0.1698	0.2369	0.8120	585	0.0004	0.5219	0.0000	780	0.0019	0.0008	*
480	0.1396	0.1977	0.7960	590	0.0008	0.4738	0.0000	790	0.0016	0.0004	*
485	0.1095	0.1711	0.7920	595	0.0005	0.4288	0.0000	800	0.0012	0.0011	*
490	0.0843	0.1629	0.7680	600	0.0006	0.3864	0.0000				
495	0.0691	0.1772	0.7100	605	0.0008	0.3491	0.0000				
